# Dual-band optical collimator based on deep-learning designed, fabrication-friendly metasurfaces

**DOI:** 10.1515/nanoph-2023-0329

**Published:** 2023-07-28

**Authors:** Akira Ueno, Hung-I Lin, Fan Yang, Sensong An, Louis Martin-Monier, Mikhail Y. Shalaginov, Tian Gu, Juejun Hu

**Affiliations:** Department of Materials Science and Engineering, Massachusetts Institute of Technology, Cambridge, MA 02139, USA; Innovative Technology Laboratories, AGC Inc., Yokohama, Japan; 2Pi Inc., Cambridge, MA, USA; Materials Research Laboratory, Massachusetts Institute of Technology, Cambridge, MA 02139, USA

**Keywords:** deep learning, fabrication tolerance, metasurface, multiband, predictive neural network

## Abstract

Metasurfaces, which consist of arrays of ultrathin planar nanostructures (also known as “meta-atoms”), offer immense potential for use in high-performance optical devices through the precise manipulation of electromagnetic waves with subwavelength spatial resolution. However, designing meta-atom structures that simultaneously meet multiple functional requirements (e.g., for multiband or multiangle operation) is an arduous task that poses a significant design burden. Therefore, it is essential to establish a robust method for producing intricate meta-atom structures as functional devices. To address this issue, we developed a rapid construction method for a multifunctional and fabrication-friendly meta-atom library using deep neural networks coupled with a meta-atom selector that accounts for realistic fabrication constraints. To validate the proposed method, we successfully applied the approach to experimentally demonstrate a dual-band metasurface collimator based on complex free-form meta-atoms. Our results qualify the proposed method as an efficient and reliable solution for designing complex meta-atom structures in high-performance optical device implementations.

## 1 Introduction

Metasurfaces are two-dimensional versions of metamaterials that provide a new platform for realizing compact, large-area optical devices and components [1–17]. By controlling the geometry of each unit cell (i.e., meta-atom), the phase, amplitude, and/or polarization of optical wavefronts can be fully engineered at the sub-wavelength scale to provide on-demand control of light propagation. One of the critical challenges in the field of metasurface design is the complexity of determining the optimal meta-atom geometries that meet a set of concurrent functional requirements, for example, optical responses at multiple wavelengths while considering manufacturability. Traditional methods use a design flow that exhausts the design space and selects results that satisfy the design requirements. The number of design degrees of freedom (DOF) determine the design time and computational resources needed. Therefore, the design often proceeds with meta-atoms limited number of DOF, such as cylinders and rectangles, in order to mitigate the computational overhead.

To enable the design of meta-atoms with complex geometries, multiparameter optimization algorithms, adjoint methods, and inverse design approaches based on neural networks have been widely studied to accommodate a large number of design degrees of freedom in order to fulfill multifunctional design specifications. In particular, deep neural network (DNN)-based data-driven modeling tools [18–21] have emerged as a powerful and versatile design approach [22–38]. Previous studies utilized fully connected layers (FCL) to precisely predict the spectral responses of bulk layers, cylinders, elliptical columns, spheres, plasmonic structures with rod-shaped meta-atoms, and all-dielectric structures [39–43]. Properly trained DNN models have demonstrated the capability to generate highly precise electromagnetic (EM) responses of free-form meta-atom structures, enabling a fast, on-demand metasurface design approach. However, relatively few examples of experimental implementations have been realized based on metasurface devices designed using these methods [44–46]. Manufacturability based on realistic fabrication constraints could be compromised given the complex geometries of meta-atoms derived from these DNN-based schemes and, therefore, must be properly considered for successful experimental demonstrations [47–52].

**Figure 1:**
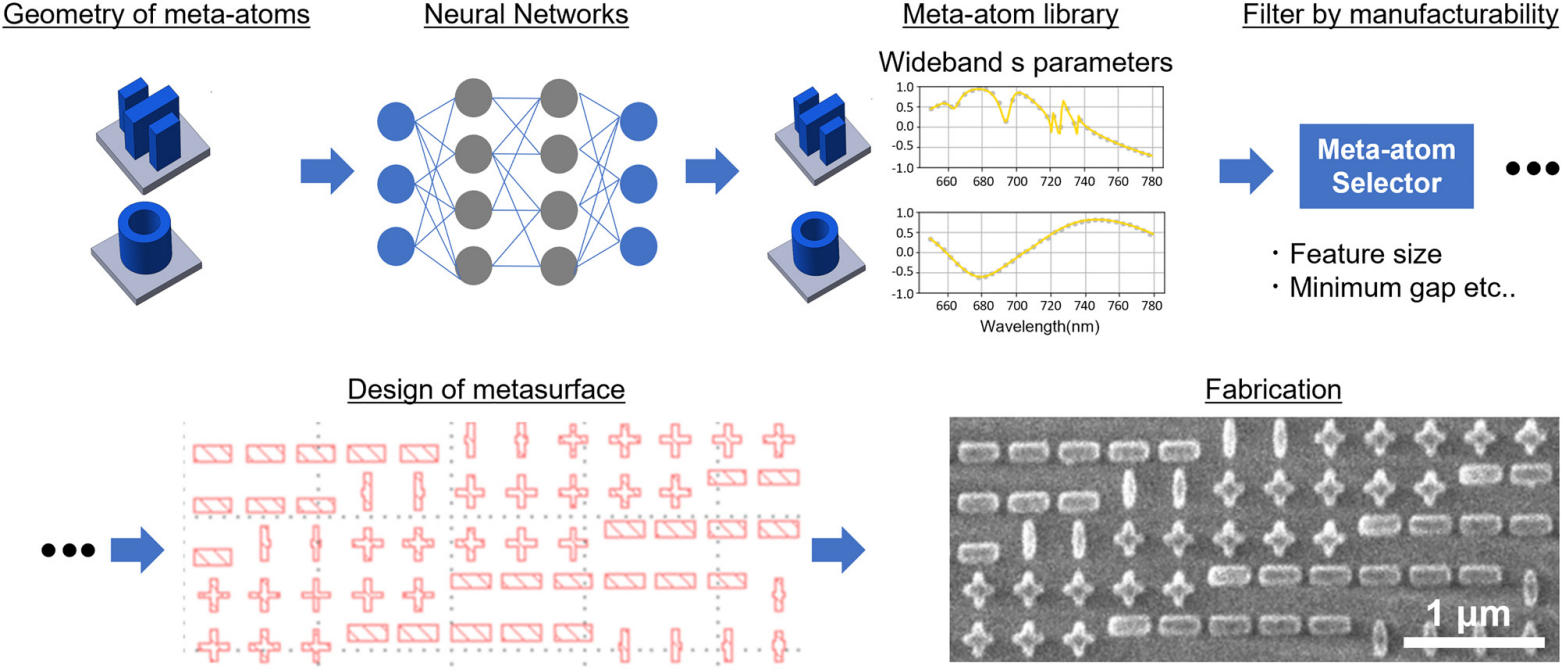
Concept of a fabrication-friendly metasurface design via machine learning. The optical responses of the meta-atom are predicted from their 2D images using a deep-learning algorithm developed to create an extensive library of meta-atoms. With this deep-learning system, the broadband optical response (e.g., amplitude and phase) of complex meta-atoms can be rapidly acquired without resorting to full-wave electromagnetic field simulations. Thereafter, meta-atoms with shapes that can be manufactured from the meta-atom library are selected using the meta-atom selector developed in this study. Using these selected meta-atoms makes it possible to design a highly functional metasurface consisting of meta-atoms with complex shapes.

In this study, we experimentally demonstrate a dual-band metasurface collimator based on DNN-designed meta-atoms. Our approach considers high-DOF meta-atom structures and imposes fabrication constraints, such as feature aspect ratio and minimum dimensions to ensure manufacturability of the design. The meta-atom library constructed by our approach improves the design cost and burden of other nonintuitive metasurface device designs that require multifunctionality and manufacturability (Figure 1).

## 2 Meta-atom design

**Figure 2:**
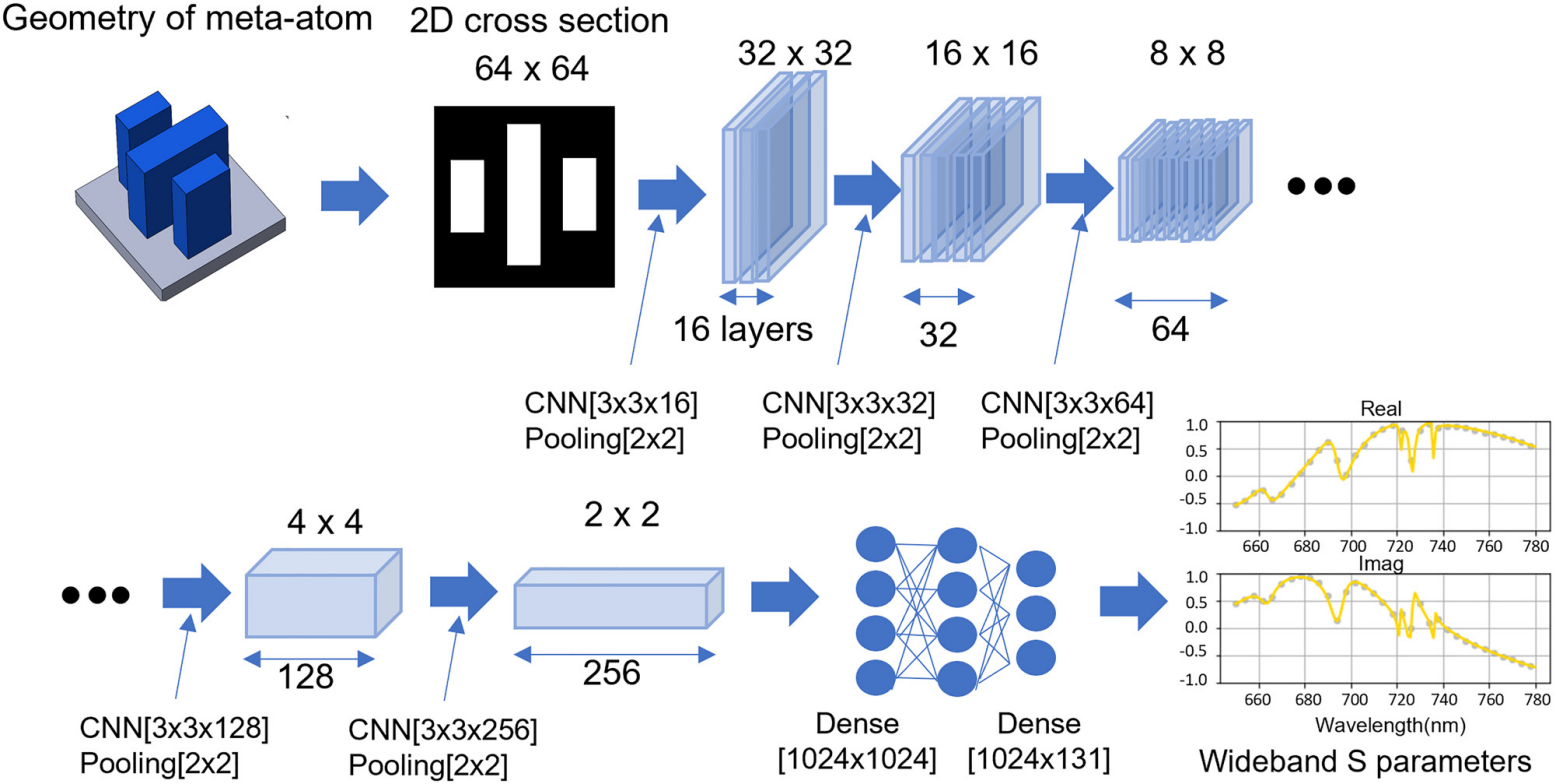
Network structure for generating meta-atom libraries. The meta-atom geometries (64 × 64 pixel 2D images) were firstly transformed with six convolution and pooling layers into a 1D array (1 × 1024) and secondly processed with three FCLs. The output real/imaginary parts (1 × 131) of the transmission coefficient over the 650–780 nm spectrum were generated for evaluation. Every convolution layer in the network was followed by a batch normalization layer.

The meta-atom library used in this study was generated using a predictive neural network (PNN) based on a convolutional neural network (CNN) architecture, as shown in Figure 2 [53]. The objective of the PNN is to predict the phase and amplitude responses of a given meta-atom design. The meta-atom model consists of quasi-free-form dielectric structures having a high refractive index on a low-index dielectric substrate forming a square unit cell. A two-dimensional pattern of each meta-atom structure (in this case, an image consisting of 64 × 64 pixels) is used as the input to the PNN and processed in the convolution and pooling layers. After flattening the output of the CNN and passing it through three fully coupled layers, the real and imaginary part predictions of the transmission coefficients are generated, which converts to phase and amplitude responses. The spectral range of interest is between 650 and 780 nm wavelengths.

Approximately 20,000 random meta-atom patterns were generated from reference structures like rectangles, crosses, hollow squares, and so on with the numerical computing tool MATLAB. Several sides of each structure were varied with a resolution of 6 nm. Then, the structures in one quadrant were symmetrically replicated along the *x* and *y* axes to form the whole pattern (Supporting Information I). Other parameters were set as follows: thickness = 490 nm, meta-atom period = 384 nm, refractive index = 3.68 to 3.93, which were based on experimentally measured values in a-Si films deposited using plasma enhanced chemical vapor deposition (PECVD) on 200 mm silica wafers from a commercial foundry. The measured refractive indices were set as a simulation condition according to the wavelength of the incident light. The electromagnetic responses of the meta-atoms were calculated using the finite difference time domain method (FDTD)-based simulation tool Lumerical as labels. The meta-atoms were randomly divided into training and test datasets, with 80 % used for training and the remaining 20 % used to evaluate the trained network. The spectral response predictions generated by the PNN were compared with the labels, and errors were extracted and minimized in the training process. After completion of DNN training, low mean squared errors (MSE) of the real and imaginary parts of the predicted transmission coefficients down to 0.0085 and 0.0085, respectively, were achieved in the test data (Supporting Information II).

**Figure 3:**
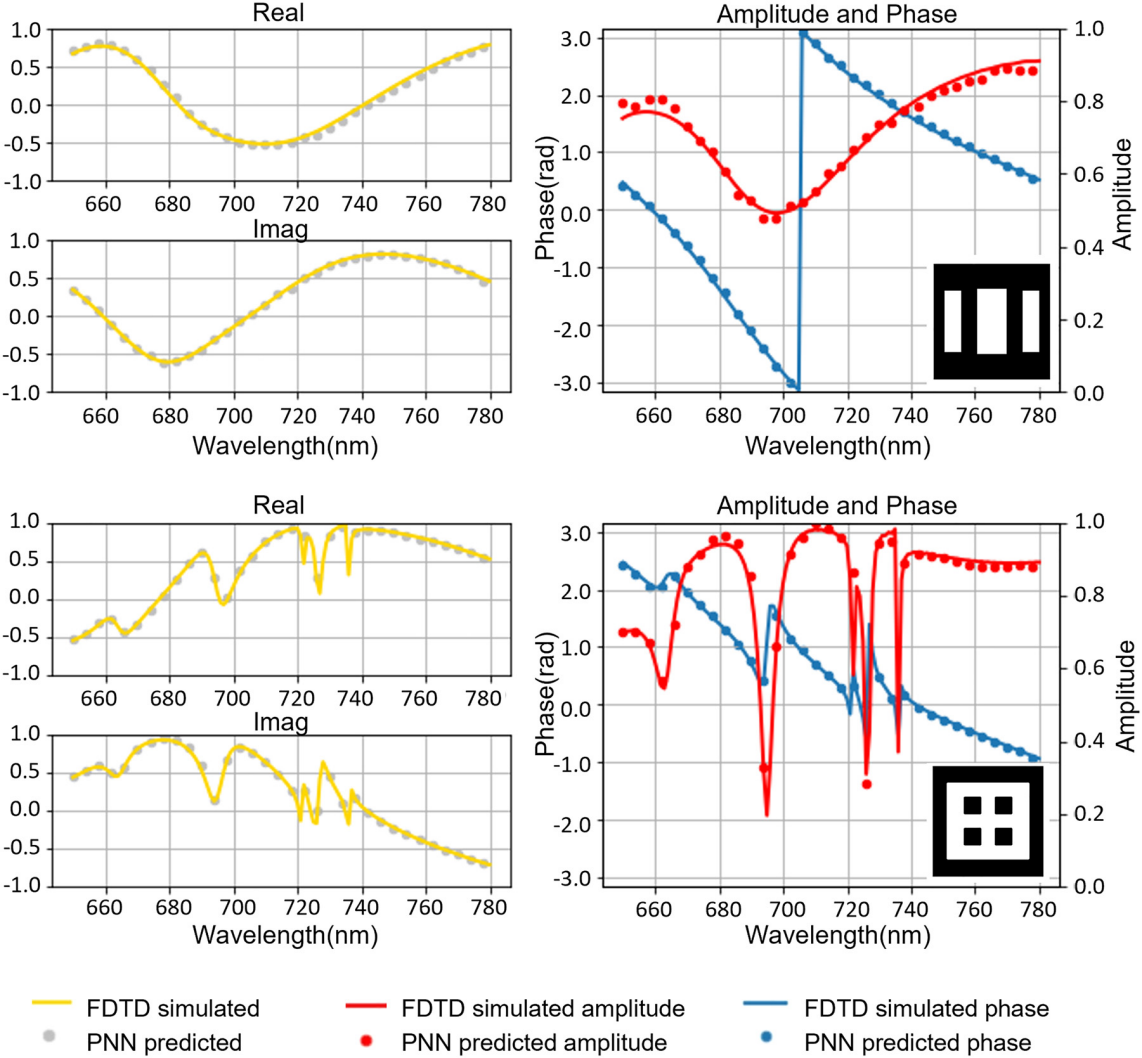
Examples of PNN predictions compared with full-wave simulation results. Small subplots shown on the left are the real and imaginary parts of transmission coefficient each meta-atoms selected from the test dataset. The right figure shows phase, amplitude prediction, and meta-atom structure. The lines indicate the PNN predictions, and the dots represent the results of FDTD simulations.

Two randomly selected meta-atom samples were obtained from the test dataset to demonstrate the effectiveness of the trained PNN. Their transmission amplitudes (red dots) and phase responses (blue dots) were evaluated and compared with the results obtained from the FDTD-based simulations (red and red-blue), as shown in Figure 3. As observed from the small test errors, the predicted results of the PNN were in good agreement with those of the full-wave electromagnetic simulations.

**Figure 4:**
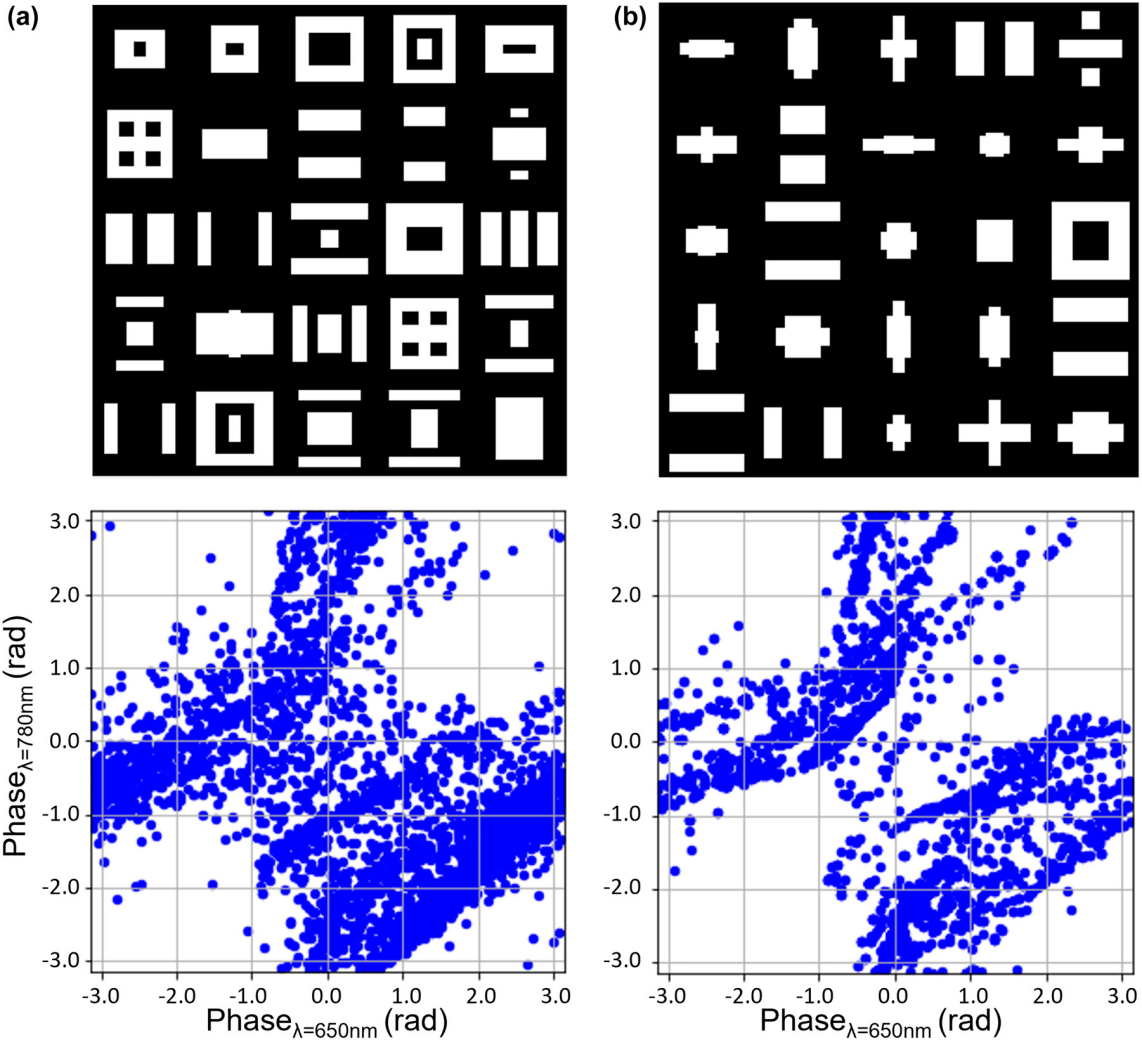
Phase coverages with PNN-generated meta-atom patterns: 3000 randomly selected patterns (a) from the complete 80,000 meta-atom library and (b) from the 5300 meta-atom library preselected with the meta-atom selector.

The fully trained PNN and 2-D image generator were used to construct a pool of meta-atoms of 80,000 free-form shapes. The pool has been obtained within only 91.7 s. Figure 4(a) shows the phase range of the meta-atom library at 650 nm and 780 nm, indicating that it covers full 2π range at both wavelengths.

The actual device fabrication requires a suitable selection of meta-atoms from the pool of meta-atoms considering the processing constraints of the fabrication method. In this study, a meta-atom selector was developed to perform the selection process. As constraints, thresholds were set for the minimum size of each structure in a unit cell, the minimum gap in a unit cell, and the gap between the unit cells. These thresholds were taken to be 60 nm as determined based on actual experimental characterizations. Based on these meta-atom designs filtered by these constraints, we can design fabrication-friendly metasurfaces. Using this meta-atom selector, 5300 patterns were selected from the library. Figure 4(b) shows the phase coverage after the selection process. We also compare the phase coverage attained by meta-atoms of simple regular geometries with the same parameters otherwise and show that the increased DOF allows the free-form meta-atoms to attain wider phase coverage, even after imposing the fabrication constraints (Supporting Information III).

Next, we discuss how we choose the meta-atoms from this library to enable dual-band operation with optimal performance. A dual-band collimator was designed and fabricated using the filtered meta-atoms library created in the previous section, and its performance was experimentally evaluated. The design principle of a hyperbolic metasurface collimating at a single wavelength has been previously reported [54, 55]. In multiband metasurface, a desired phase delay: *φ*_*λ*,*r*_ at a given radial position *r* with respect to the center of the array and at each wavelength *λ*_1_ and *λ*_2_ is given by:

$$\varphi_{\lambda 1,r}=-\frac{2\pi n}{\lambda_{1}}\left(\sqrt{r^{2}+f^{2}}-f\right)$$

$$\varphi_{\lambda 2,r}=-\frac{2\pi n}{\lambda_{2}}\left(\sqrt{r^{2}+f^{2}}-f\right)$$

where *f* is the focal length and *n* is the refractive index of the substrate (for a glass substrate at the wavelengths of interest, material dispersion has been neglected). The collimator diameter is 1 mm and the focal length is 15 mm in our design. The metasurface layout is generated based on the phase masks and individual meta-atom responses at the two wavelengths, using a design figure of merit (FOM) tailored for multifunctional metasurfaces, which allows evaluating and optimizing meta-atom designs without resorting to full-scale system simulation [56]. Meta-atoms were selected from the design pool based on the following FOMs:

$$FOM_{\lambda 1,r}=T_{\text{meta}.\lambda 1}\cdot \left(\frac{sin\left(2\left|\varphi_{\text{meta},\lambda 1}-\varphi_{\lambda 1,r}\right|\right)}{2\left|\varphi_{\text{meta},\lambda 1}-\varphi_{\lambda 1,r}\right|}\right)$$

$$FOM_{\lambda 2,r}=T_{meta,\lambda 2}\cdot \left(\frac{\sin\left(2\left|\varphi_{meta,\lambda 2}-\varphi_{\lambda 2,r}\right|\right)}{2\left|\varphi_{meta,\lambda 2}-\varphi_{\lambda 2,r}\right|}\right)$$

$$FOM_{\text{eff}}=\sqrt{FOM_{\lambda 1,r}\cdot FOM_{\lambda 2,r}}$$

where FOM_*λ*1,*r*_ and FOM_*λ*2,*r*_ are correlated with metasurface performance at the two wavelengths. *T*_meta,*λ*_, *φ*_*λ*,*r*_, and *φ*_meta,*λ*_ are the meta-atom transmission, target phase delay value imparted by the meta-atom, and actual meta-atom phase value, respectively. By maximizing the FOM, the tradeoff between transmission and phase errors can be balanced quantitatively. This enables the synthesis of metasurfaces with optimal meta-atom structures without performing full-scale simulations of the entire optical system. For each meta-atom position across the aperture, the FOM is calculated based on the local phase values at the two bands, and the optimal meta-atom selection is made to fill the position. The process is repeated across the aperture to generate the final metasurface design. The calculated FOMs in our design were 0.688 at 650 nm and 0.768 at 780 nm. Furthermore, we estimated the transmission efficiency using the Kirchhoff diffraction integral [57], which allows us to transform the near-field wavefront after exiting the metasurface to the intensity distribution at the image plane. The estimated efficiencies at 50 mm from the metasurface were 0.609 at 650 nm and 0.742 at 780 nm.

## 3 Metasurface fabrication and characterization

**Figure 5:**
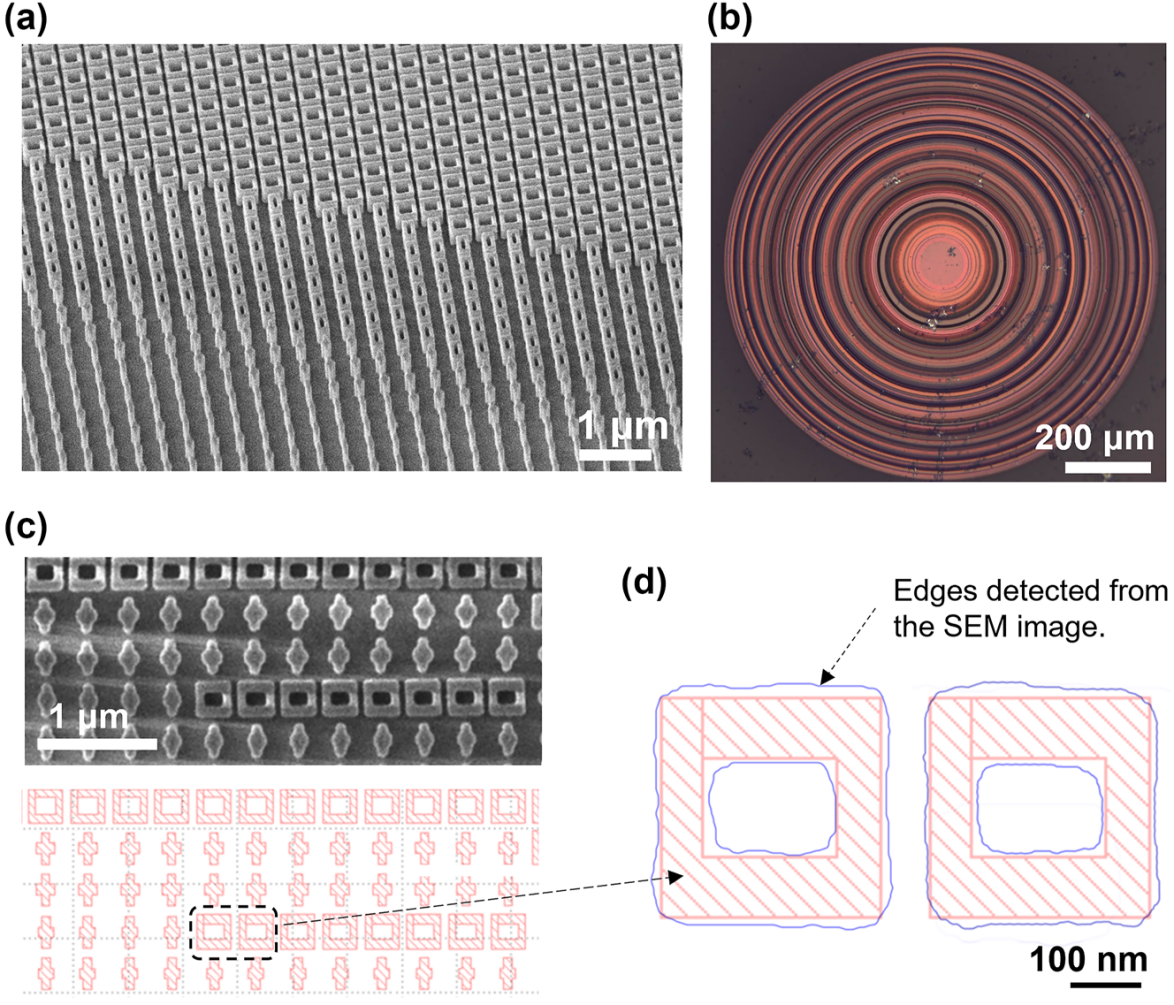
Fabricated metasurface. (a) SEM image at a wide viewing angle. (b) Optical microscope image. (c) SEM image of meta-atom array alongside corresponding GDS layout of the same area. (d) Comparison of edges contours retrieved from the SEM image in (c) (shown in blue line) and GDS layout (shown in red area).

The metasurfaces were fabricated using electron-beam lithography and plasma etching. Details of the fabrication process are described in Section 5. Figure 5(a) and (c) shows the scanning electron microscopy (SEM) images of the fabricated metasurface in comparison with the design. To quantitatively assess the pattern fidelity, we used the image processing software MauntainsSEM to extract the contours representing the edges of the meta-atoms and compare them to the GDS (Graphic Data System file format) mask design. As an example, Figure 5(d) presents a chart that overlays the meta-atom contour obtained from the SEM image with the mask layout. For this type of “hollow square” meta-atom, we measured an average size deviation of 8.3 nm and corner rounding with an average radius of 16.7 nm. We further feed the experimentally fabricated meta-atom geometry to the PNN and found that the deviation has a negligible impact on the meta-atom phase and transmittance (Supporting Information IV). A metal aperture was patterned on the substrates to block optical transmission from areas not covered by the metasurface to eliminate unwanted stray light (Figure 5(b)).

**Figure 6:**
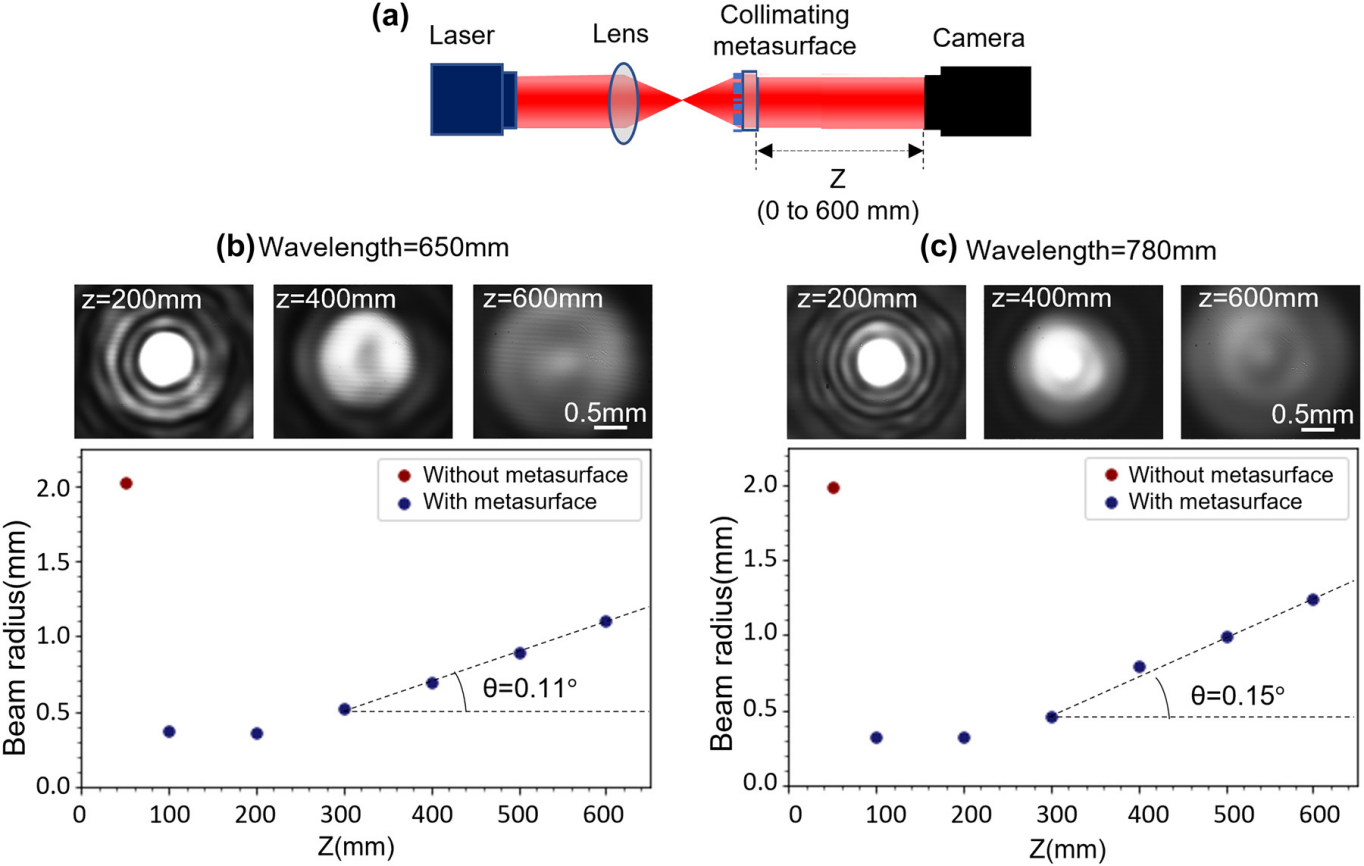
Characterization of metalens-collimated laser beams. (a) Schematic illustration of the measurement setup. Collimation performance with and without metasurface at (b) 650 nm and (c) 780 nm wavelengths.

A schematic of the characterization setup for the beam profiles is shown in Figure 6(a). Collimated semiconductor lasers (Thorlabs SM Fiber-Pigtailed Laser Diode) emitting at 650 nm and 780 nm wavelengths were used as light sources. The laser beam was focused and diverged by a lens (Thorlabs N-BK7 plano-convex lense) with NA equal to the NA of the designed metasurface collimator. Light passing through the metasurface was captured using a CMOS image sensor (Arducam 10 MP MT9J001 Monochrome CMOS 1/2.3″ Camera Module). The distance between the CMOS image sensor and the metasurface was varied from 0 to 600 mm using a linear translation stage, and the divergence of the beam diameter (defined as a width at 1/*e*^2^ of the peak intensity using MATLAB) was recorded. A substrate with a clear 1 mm diameter aperture was used as a reference.

Figure 6(b) plots the measured beam diameter distributions along the propagation direction with and without the metasurface collimator at 650 nm wavelength. Laser propagation without the metasurface collimator results in steep divergence of the outcoming light. In contrast, beam exiting from the metasurface collimator maintains a stable size. The divergence angle of the metasurface-collimated beam was 0.11°. Similar behavior was measured at 780 nm wavelength as well with a small divergence angle of 0.15°, as shown in Figure 6(c). The total transmission efficiency measured at 50 mm from the metasurface were 50.3 % at 650 nm and 56.8 % at 780 nm. The result demonstrates viability of using free-form meta-atoms designed by DNN to realize multifunctional phase-gradient metasurface devices.

## 4 Conclusions

In this study, we developed a deep-learning–enabled, fabrication-friendly metasurface design framework and experimentally demonstrated a dual-band metasurface collimator operating at 650 nm and 780 nm wavelengths concurrently. The metasurface was designed using a free-form meta-atom library generated from DNN with imposed fabrication constraints and a simple FOM to optimize the dual-wavelength meta-atom arraying without having to perform full-device simulations. The processability-aware DNN-based method proposed in this study is a fast and accurate modeling tool that provides a practical link between an extensive and sophisticated parametric space and the corresponding physical response. The approach can be applied to realize a wide variety of innovative multifunctional optical devices.

## 5 Experimental section

Metalens fabrication: A 490 nm thick amorphous Si film was deposited on a 200 mm fused silica wafer by plasma-enhanced chemical vapor deposition by a commercial foundry. The wafer was then diced into square pieces with a side length of 12.5 mm as the metasurface substrates. To fabricate the mask patterns, a negative tone of electron beam resist (ma-N 2402 from a mixture of ma-N 2401 and ma-N 2403, Micro Resist Technology) and a conductive polymer (ESpacer 300Z, Showa Denko America, Inc.) were spin coated onto substrates for electron-beam lithography (EBL). The use of a conductive polymer prevents charging effects during EBL writing. The EBL was conducted at a voltage of 50 kV and a beam current of 1 nA on an Elionix HS50 system. The sample was then placed in a developer (AZ 726 MIF Developer) to produce the resist mask patterns and gently rinsed with deionized water. To etch the amorphous Si, dry etching was performed using dual plasma sources and dual gas inlets with a mixture of SF_6_ and C_4_F_8_ (SPTS Rapier DRIE). The residual electron-beam resist was stripped using O_2_ plasma ashing. The areas on the substrate that were not occupied by the metasurface were subsequently covered with a metal mask to prevent stray light. To fabricate the metal mask, a negative-tone photoresist (AZ nLOF 2035) was spin coated onto the metasurface at 2500 rpm. The resist was soft baked at 115 °C for 1 min, exposed to UV light on a MLA150 Maskless Aligner, and then baked at 110 °C for 1 min. The photoresist was developed by immersing the sample in a Microposit MF-319 developer for 1 min. Then, a 120 nm thick Au layer was deposited by electron beam evaporation at a rate of 2.0 Å/s in a Sharon electron beam evaporator. Finally, the photoresist was removed using a solvent stripper (Remover PG, MicroChem) to pattern the metal mask via lift-off.

## Supplementary Material

Supplementary Material Details
