# Supplementary material for: Dual-band optical collimator based on deep-learning designed, fabrication-friendly metasurfaces
Source: Nanophotonics. 2023 Jul 28;12(17):3491–9. doi: 10.1515/nanoph-2023-0329 (PMC11501907; doi:10.1515/nanoph-2023-0329)
Supplement: Supplementary file 1 — Supplementary Material Details [file j_nanoph-2023-0329_suppl_001.pdf]

Supporting Information

Deep learning modeling approach for a high-functionality and fabrication-friendly metasurface

Akira Ueno[1,2], Hung-I Lin[1], Fan Yang[1], Sensong An[1], Louis Martin-Monier[1], Mikhail Y. Shalanginov[1], Tian Gu[1,3] and Juejun Hu[1]

[1]Department of Materials Science & Engineering, Massachusetts Institute of Technology, Cambridge, Massachusetts 02139, USA

[2]AGC Inc., Yokohama, Kanagawa 230-0045, Japan

[3]Materials Research Laboratory, Massachusetts Institute of Technology, Cambridge, Massachusetts 02139, USA

In this Supporting Information, we provide further information that our DNNs methods and examples.

I. Meta-atom design for DNNs training
II. Hyperparameters used in the training of DNNs
III. Additional DNN prediction examples
IV. The estimation of influence of the fabrication deviation for meta-atom performance.

Section I – Meta-atom design for DNNs training

Without loss of generality, the all-dielectric meta-atom comprises a 490 nm a-Si layer sitting on a fused silica substrate, with a unit cell size of 384 × 384 nm^2 as shown in Fig.S1a. The 2D pattern of each meta-atom was generated using the parametric approach, referring to the Fig. S1b patterns, with the numerical computing tool MATLAB. Several sides of each structure were varied with a resolution of 6 nm. Then, the all-dielectric components were symmetrically replicated along the x and y axes to form the whole pattern. A set of meta-atoms generated in this manner is guaranteed to possess polarization-diverse performance. The full-wave electromagnetic simulations were performed using the commercial FDTD simulation tool Lumerical. For each meta-atom, perfect electric conducting surface (Et = 0) and perfect magnetic conducting surface (Ht = 0) boundary conditions were employed to calculate the transmission and phase shift of a square lattice structure. Open boundaries were applied along both the negative and positive z directions, while a plane wave was illuminated from the substrate side for each meta-atom. To further accelerate the full-wave simulations, Et = 0 and Ht = 0 symmetry planes were applied in the center y-z and x-z planes for each meta-atom, respectively. Twenty thousand meta-atoms with different shapes were generated and simulated to determine their wide-spectrum phase and amplitude responses. These simulations were performed on two servers running in parallel. The data collection process was completed in 14 days.

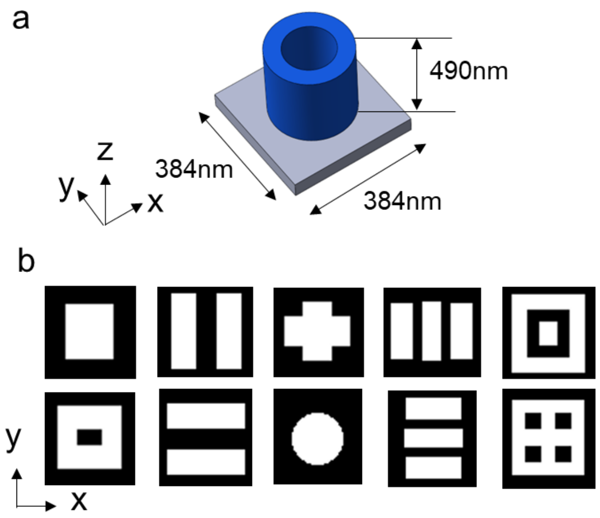

Fig. S1. (a) Three-dimensional view of the meta-atom designed for training deep neural networks (DNNs). The unit cell area is fixed to be 384 nm x 384 nm, and the height is 490 nm. (b) Base-10 representations of the meta-atom patterns.

Section II – Hyperparameters used in the training of forward prediction DNNs

The hyperparameters utilized in training for the forward prediction DNN is displayed in Table S1. The hardware setup consists of a 16-core CPU with a clock speed of 5.0 GHz, 128 gigabytes of RAM, and NVidia 3080 GPU. As demonstrated in the table, the average test set error stabilized at 0.0085 and 0.0085 for the real and imaginary parts, respectively, for the forward prediction networks after 500 iterations. Using the current hardware configuration, the training of both DNNs takes 5.5 hours before their error rates become stable. The learning curves, which record the error history during the training of each network, are shown in Fig. S2.

Table S1. Hyperparameters used in the training of forward prediction DNNs

**Hyperparameters**	**Forward DNN (real)**	**Forward DNN (Imaginary)**
**Training set size**	*16,000*	*16,000*
**Test set size**	*4,000*	*4,000*
**Optimizer**	Adam	Adam
**Learning rate**	10^{-4}	10^{-4}
**Batch size**	*32*	*32*
**Nonlinear activations**	ReLU	ReLU
**Iterations**	*500*	*500*
**Time taken**	*5.5h*	*5.5 h*
**Error**	*0.00165 (0.00846)*	*0.00147 (0.00848)*

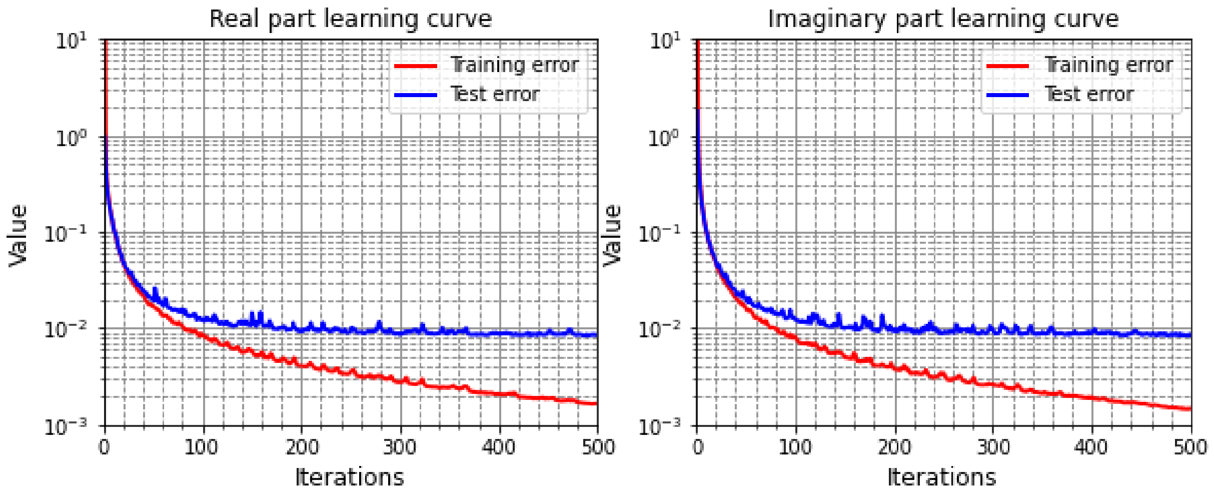

Fig.S2. Learning curves of the forward DNNs. Showing in the figures are the learning curves of real part DNN; imaginary part DNN.

Section III – Additional DNN prediction examples

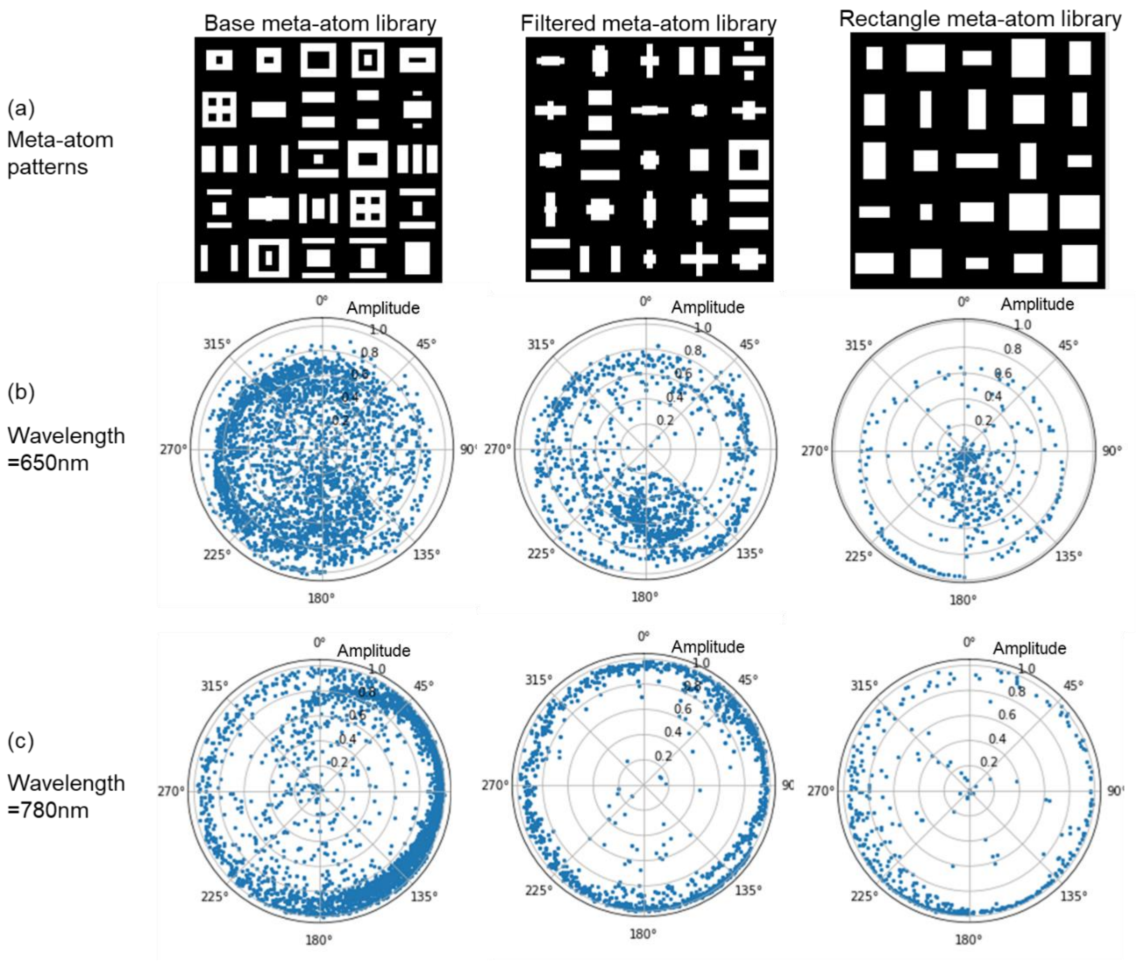

Fig. S3. Comparing the performance of meta-atoms with different shapes using the DNNs. (a) Examples of randomly-generated 25 patterns for base meta-atom library, filtered meta-atom library and rectangle library. (b)Transmissive responses of meta-atoms from the respective libraries at the wavelengths of (b) 650 nm and (c) 780 nm.

Section IV – The estimation of influence of the fabrication deviation for meta-atom performance.

Using the PNN, we demonstrate the estimation of the transmission drop and phase error of the size deviations shown in Fig.5(d). In comparison with the designed value, the simulated value that uses actual dimensions shows a 6.1% transmittance drop, a 2.2° phase error at 650nm and a 1.9% transmittance drop, a 0.2° phase error at 780nm.

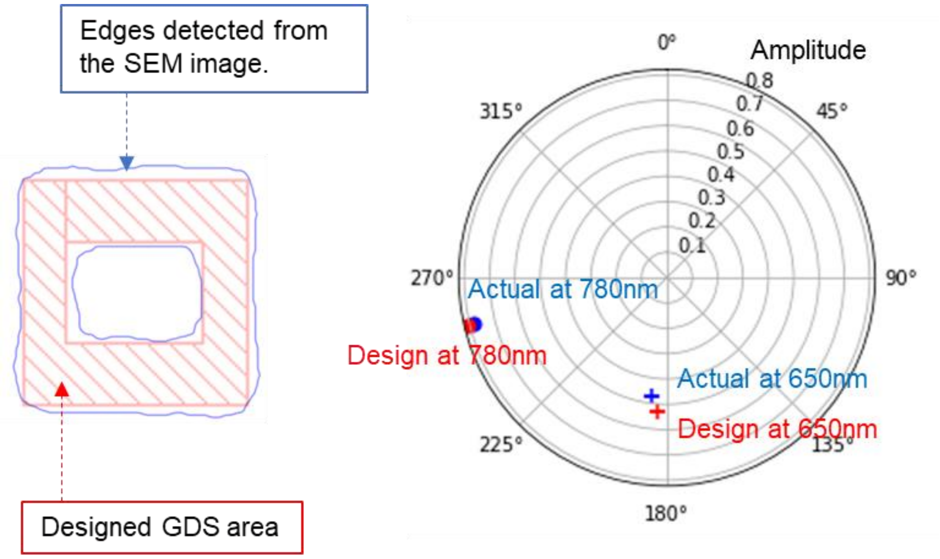

Fig.S4. Comparing the performance of meta-atoms with designed shape and actual fabricated shape.
